# Arthrofibrosis after Anterior Cruciate Ligament Reconstruction: The Devil is in the Detail

**DOI:** 10.5704/MOJ.2011.036

**Published:** 2020-11

**Authors:** RY Kow, CL Low

**Affiliations:** 1 Department of Orthopaedics, Traumatology and Rehabilitation, International Islamic University Malaysia (IIUM), Kuantan, Malaysia; 2 Department of Radiology, International Islamic University Malaysia (IIUM), Kuantan, Malaysia

Dear editor,

We read with great interest the article by Rushdi *et al* in the November 2019 issue entitled “Arthrofibrosis Following Anterior Cruciate Ligament Reconstruction”^1^. In their paper, they review the complications of arthroscopic anterior cruciate ligament (ACL) reconstruction at two centers, with special emphasis on arthrofibrosis and its predisposing factors. While ACL reconstruction is one of the most commonly performed orthopaedic procedures in the world, the reported outcome of ACL reconstruction and its complications are scarce in Malaysia^2^. For this reason, authors should be applauded for their effort.

Nevertheless, there are a few important issues which should be addressed in this article. First and foremost, it is a relatively short duration of 6 months to assess the outcome of ACL reconstruction. In their study, authors claim that preoperative limited motion, timing of surgery and female patients are correlated with arthrofibrosis, despite offering no data analysis to support this claim. Authors postulate that female patients are at a higher risk (16%) to develop arthrofibrosis after ACL reconstruction as compared to male counterpart (4%). With such a disproportionate male-to-female ratio (only 7% female included) in this study, any result must be interpreted with caution, preferably with univariate or even multivariate analysis. Similarly, the claim that patients who undergo earlier surgery is at a higher risk of arthrofibrosis does not hold water as there is only one patient who has undergone surgery at two weeks post-injury. For those who develop arthrofibrosis, five of them (62.5%) require arthroscopic arthrolysis at three months after ACL reconstruction. It is worth contemplating whether a short duration of follow-up (three months) after an arthroscopic arthrolysis is adequate in labelling those patients “healed”.

While authors should be commended for sharing their results, we are afraid that the article has wasted a chance to be more meaningful by not adding more information and analysis.

## References

[1] Rushdi I, Sharifudin S, Shukur A (2019). Arthrofibrosis following anterior cruciate ligament reconstruction. Malays Orthop J..

[2] Tengku Muzaffar TMS, Shahrulazua A, Musa K, Masdiamin MN, Fuad DM, Amiruddin HM (2013). The activity leading to ACL injury and the ability to resume duty following reconstructive surgery in Malaysian military patients. Med J Malaysia..

