# Supplementary material for: Arthrofibrosis after Anterior Cruciate Ligament Reconstruction: The Devil is in the Detail
Source: Malays Orthop J. 2020 Nov;14(3):204–5. doi: 10.5704/MOJ.2011.036 (PMC7751994; doi:10.5704/MOJ.2011.036)
Supplement: Related texts

# Reply: Response to Letter to Editor

**Published:** 2020-11

**Authors:** Rushdi Isnin

**Affiliations:** Corresponding author, Department of Orthopaedics, Hospital Teluk Intan, Teluk, Intan, Malaysia

Dear Dr,

Thank you for your letter to editor with title ‘Arthrofibrosis After ACL Reconstruction: The Devil is in the Detail’ regarding the article by Rushdi *et al* in the November 2019 issue entitled “Arthrofibrosis Following Anterior Cruciate Ligament Reconstruction”^1^.

We would like to give you our warmest regards regarding this letter to editor and thank you for your appraisal regarding our effort. Firstly, we would like to enlighten that our study was mainly an observational study of case series on arthrofibrosis and its predisposing factors. This means that it is a descriptive study through data collection retrospectively and analysis using pre-existing, pre-operative limited motion^2,3^ and female incidence^2^. The limitation of this study is relatively small sample size which we couldn’t get sufficient samples to represent the population and does not allow us to do univariate and multivariate analysis due to inadequate power.

The issue that was questioned in the letter was regarding the relatively short duration of six months to assess the outcome of ACL reconstruction. We would like to emphasise that our study aims towards arthrofibrosis specifically^1^. Ideally, arthrofibrosis should be detected during early post-surgery follow up. Delay in detecting arthrofibrosis will cause delayed in management causing undesirable outcome4. We would like to clarify that all our patients were followed up more than six months and not three months as stated in your letter. In our study, three months is the period when we proceed with arthroscopic adhesiolysis procedure. This does not label patient as ‘healed’ as stated in the letter.

Regarding time of surgeries, Quelard *et al*^2^, Mayr *et al*^3^, Shelbourne *et al*^4^, Wasilewski *et al*^5^, Johnson *et al*^6^ reported similar findings in which they found majority of arthrofibrosis occurred if the surgeries were done immediately after an acute knee injury, and post-operative motion recovery was significantly less comparing to those who have delayed surgery. Lastly, we hope that our findings will initiate more research to be conducted in this field. Ideally, a prospective and multi-centre study with larger sample size to represent our Malaysia population.

Thank you.

## References

[1] Rushdi I, Sharifudin S, Shukur A (2019). Arthrofibrosis following anterior cruciate ligament reconstruction. Malays Orthop J..

[2] Quelard B, Sonnery-Cottet B, Zayni R, Ogassawara R, Prost T, Chambat P (2010). Preoperative factors correlating with prolonged range of motion deficit after anterior cruciate ligament reconstruction. Am J Sports Med..

[3] Mayr HO, Weig TG, Plitz W (2004). Arthrofibrosis following ACL reconstruction--reasons and outcome. Arch Orthop Trauma Surg..

[4] Shelbourne KD, Payel DV, Martini DJ (1996). Classification and management of arthrofibrosis of the knee after anterior cruciate ligament reconstruction. Am J Sports Med..

[5] Wasilewski SA, Covall DJ, Cohen S (1993). Effect of surgical timing on recovery and associated injuries after anterior cruciate ligament reconstruction. Am J Sports Med..

[6] Johnson DL, Bealle DP, Brand JC Jr, Nyland J, Caborn DN (2000). The effect of a geographic lateral bone bruise on knee inflammationafter acute anterior cruciate ligament rupture. Am J Sports Med..
